# The Poor Man's Home.—XXVII

**Published:** 1898-02-26

**Authors:** 


### MODERN SOCIOLOGY.

### THE POOR MAN'S HOME.—XXVII.

### BUILDING SOCIETIES.

WHEN first public attention was directed to the high rents that the poor were forced to pay the popular remedy was the institution of societies by which a man with little or no capital could become the owner of his own house. The primary aim of these societies was philanthropic, while they were, as a rule, managed on sound business principles; and, in spite of frauds and failures, of which it is not necessary here to speak, they have in the main fulfilled the object for which they were instituted—they have enabled working men to become the owners of their own homes. For many men this is a boon, and we must not be understood to deny or minimise this, while we point out cases in which it is better for a working man to be only a tenant and not his own landlord.

As soon as a man is sure that he will remain in the town or village of his present residence for the whole of his life, it is better for him to build or buy a house. Even if he borrows great part of the purchase-money, the interest on this will not amount to more—on most cases it will be considerably less—than the rent of a house, and every penny which he can pay off is so much saved, and helps him to become the proprietor of the house. Moreover, the hope of becoming one's own landlord is a great inducement to thrift. Add to this, that every man has his own special fancies and requirements in housebuilding, and that the tenement house, built to suit the most apparent needs of the greatest number, is rarely just what anyone would have built for himself. These are the advantages of owning one's house, and it must be admitted that they are great. But they all depend on our first assumption, that the owner intends to remain in the one place all his days. To a man whose ultimate destiny is not yet fixed, a house is not a boon but a burden.

We have not fully grasped the economic importance of the mobility of labour. That working men should be able to go as freely as possible to the place where their labour is most needed is a matter of importance not only for themselves but for the whole community. In one part of the world, or even of the same country, work may be waiting for men to do it, while in another labourers may be falling into poverty for lack of employment. Therefore, it is good both for employers and employed that the latter should be able to shift their camp with as little trouble as possible. Now, a working man's worldly goods are not as a rule so great that they encumber him to a very large extent in his journeyings. But if he adds to his ordinary impedimenta of wife, children, and furniture, a house, the case is altered. Anyone who has ever sold a house knows that it is not easy to get a remunerative price for it. If the owner has time and means to hold out till he gets what he considers to be a sufficient offer, so much the better for him. But we are not thinking of such a case, which would rarely occur in the case of a working man who was leaving one town for another, in the hope of obtaining better paid employment. He feels that if he is to go at all he must go at once. Therefore, on the one hand, he must take what he can get, while on the other, he is unwilling to throw away the hard-earned money he has invested in the house by selling it for less than it cost. But the seller's extremity is the buyer's opportunity; and anyone who knows his circumstances will offer as little as he possibly can, consistently with a chance of getting what he wants. Indeed, if trade be leaving the town where our hypothetic house-owner lives, a sum much less than it was worth when he built it may be a quite sufficient payment. But in any case he cannot afford to lose the difference between the sum offered him and that which the house originally cost. It is obvious that if his money had been put in the savings bank, and the interest of it drawn to pay house rent, he would be in a much more independent position if he thought it wise to leave the neighbourhood or the country. To a man trained in business ideas this would at once be clear, but it is not easy for the average artisan to look so far ahead.

The strength of labour lies more in mobility than in trades unions or any artificial organisation, and anything that lessens that mobility weakens its power of altering its conditions. There is, indeed, in all occupations a floating population, usually single men, who are ready to move to another place on the slightest prospect of better wages; and this is so far good that it leaves the married men, who would find such constant changing difficult, in possession of the original field of employment. But when even this does not bring the supply of labour down to a level with the demand, it is a pity that it should be those who have shown themselves the most prudent and thrifty who, through the use to which they have put those very qualities, are forced to remain in the less promising place, and are as little able to take the opportunity of going elsewhere and improving their position, as the most improvident of their fellows.

It is possible that these ideas are being grasped by the working classes, for we do not think that the building society, with its advice to be one's own landlord and pay no rent, is quite as popular among that class as it used to be. But the distrust may be due simply to the bad management that has marked some of these societies, and the fraud that has stained the record of others. Of course, when, as in the generous scheme of Mr. Cadbury, which we spoke of in an earlier article, there is an undertaking to buy the house at a fair price in the case of the occupant wishing to dispose of it, it is another matter; but, in ordinary circumstances, anyone who goes to the expense of building a house should be fairly sure that he will remain in the same place a sufficiently long time to make it worth his while to risk some loss when he or his children come to realise it.

In this country the builders of blocks or cottages for the working people do not, as a rule, press them to purchase their dwellings. In France, on the contrary, the idea of fixing the *ouvrier* in a home of his own is a favourite one with philanthropists. But the authorities at Mulhouse, where the purchase of houses is the rule, find that even in this respect the plan is not satisfactory. When the house is bought money may be borrowed from a usurer on the security of it, and, moreover, it is found to be more difficult to enforce regulations against overcrowding when the occupant is proprietor of the house than when it is the property of a landlord or association who possesses more intelligent ideas of sanitation.

